# Prevalence and Patterns of Multi-Morbidity in Serbian Adults: A Cross-Sectional Study

**DOI:** 10.1371/journal.pone.0148646

**Published:** 2016-02-12

**Authors:** Dragana Jovic, Dejana Vukovic, Jelena Marinkovic

**Affiliations:** 1 Center for Hygiene and Human Ecology, Institute of Public Health of Serbia, Belgrade, Serbia; 2 Institute of Social Medicine, Faculty of Medicine, University of Belgrade, Belgrade, Serbia; 3 Institute of Medical Statistics and Informatics, Faculty of Medicine, University of Belgrade, Belgrade, Serbia; University of Crete, GREECE

## Abstract

**Introduction:**

Like many developing countries, Serbia is facing a growing burden of chronic diseases. Within such public health issue, multi-morbidity requires a special attention.

**Aims:**

This study investigated the prevalence of multi-morbidity in the Serbia population and assessed the co-occurrence of chronic diseases by age and gender.

**Methods:**

We analyzed data from the 2013 National Health Survey, which included 13,103 individuals ≥ 20 years old. Multi-morbidity patterns were identified by exploratory factor analysis of data on self-reported chronic diseases, as well as data on measured body weight and height. The analysis was stratified by age and gender.

**Results:**

Multi-morbidity was present in nearly one-third of respondents (26.9%) and existed in all age groups, with the highest prevalence among individuals aged 65 years and older (47.2% of men and 65.0% of women). Six patterns of multi-morbidity were identified: non-communicable, cardio-metabolic, respiratory, cardiovascular, aggregate, and mechanical/mental/metabolic. The non-communicable pattern was observed in both genders but only in the 20–44 years age group, while the aggregate pattern occurred only in middle-aged men. Cardio-metabolic and respiratory patterns were present in all age groups. Cardiovascular and mechanical/mental/metabolic patterns showed similar presentation in both men and women.

**Conclusions:**

Multi-morbidity is a common occurrence among adults in Serbia, especially in the elderly. While several patterns may be explained by underlying pathophysiologies, some require further investigation and follow-up. Recognizing the complexity of multi-morbidity in Serbia is of great importance from both clinical and preventive perspectives given that it affects one-third of the population and may require adjustment of the healthcare system to address the needs of affected individuals.

## Introduction

Chronic diseases are among the greatest public health challenges faced by populations around the world [1]; two or more chronic diseases can co-exist in the same individual, which is referred to as multi-morbidity [2,3]. Accurate and earlier detection of diseases has improved with advances in medical technologies, healthcare, and monitoring systems; however, multi-morbidity remains a significant ongoing problem, with the number of affected patients continuing to rise [4–6], affecting individuals of all ages and both genders [7,8]. Multi-morbidity leads to reduced functioning, making effective treatment more difficult and decreasing patients’ quality of life [9]. Adjusting health care to address multiple chronic conditions is also a challenge, since clinical guidelines typically focus on a single disease, while adhering to these in cases of multi-morbidity can have adverse effects [10]. Recent studies have revealed multi-morbidity patterns consisting of clustered but distinct clinical entities, which complicate etiological research and treatment of chronic diseases [11]. To date, there have been few large-scale studies on multi-morbidity in underdeveloped and developing countries.

Serbia is situated in South-Eastern Europe; life expectancy is 72.46 years for males and 77.68 years for females, with elderly persons constituting a growing percentage of the total population (17.8% of individuals were aged 65 years and older in 2013). Accordingly, the prevalence of chronic diseases among adults in Serbia has been increasing over the last 15 years [12–14], which has also been linked to negative socioeconomic trends in the last decade of the previous century that have affected the health status of the population [13]. Serbian citizens are primarily afflicted with non-communicable diseases—including cardiac ischemia, cerebrovascular diseases, lung cancer, affective disorders (unipolar depression), and diabetes—that account for nearly two-thirds of the total disease burden [15]. Health care in Serbia includes preventive, curative, rehabilitative, inpatient, and outpatient specialist care, and primary care including medications, home care, and medical transportation [16]; this is primarily financed through mandatory contributions to the Health Insurance Fund, which guarantees access to a relatively broad package of medical services to the entire population [16]. In order to improve the quality of health care, standards for good clinical practice (or clinical guidelines) have been developed in Serbia for use by hospital and primary care clinicians. However, as elsewhere, these guidelines were developed for the treatment of a single medical condition, which does not always apply to patients with multi-morbidity. Some studies have examined the co-occurrence of chronic disease in the Serbian population [17], but none have investigated the prevalence or patterns of multi-morbidity or the demographic group that is predominantly affected. The present study addressed this by estimating the population-based prevalence of multi-morbidity in Serbia according to age and gender, and assessing the co-occurrence of chronic diseases, including their clustering by age/gender subgroups.

## Materials and Methods

### Study design and population

This study represents a secondary analysis of data from the 2013 National Health Survey (NHS 2013) of the Serbian population (excluding Kosovo and Metohia). A stratified, two-stage representative sample of the population was selected for the survey to obtain statistically reliable estimates at the national level by examining the major geographical areas/statistical regions in Serbia, including urban and rural settlements/areas (Vojvodina, Belgrade, and Sumadija and Western, Southern, and Eastern Serbia). The units of the first stage of sampling were census enumeration areas, selected based on probability proportional to size (i.e., probability proportional sampling); a total of 670 census enumeration areas were thus selected. The units of the second stage of sampling were households selected by simple random sampling without replacement. Ten households were included in each census enumeration area along with three backup households. Out of 2,487,886 households registered by the 2011 Serbian Population Census, 10,089 were randomly selected; of these, 6,500 agreed to participate in NHS 2013, yielding a household response rate of 64.4%. Of 16,474 registered members of households who were older than 15 years, 14,623 were interviewed, corresponding to a response rate of 88.9%. All study procedures were in accordance with the European Health Research Second Wave [18–21], World Medical Association Declaration of Helsinki, Serbian regulations on personal data protection and official statistics [22,23], and Directive 95/46/EC. Information on the health of the population was gathered through a face-to-face interview using standardized questionnaires, by anthropometry, and from blood pressure measurements. Interviews and measurements were carried out in each household by teams consisting of two trained interviewers and a healthcare worker. Informed, written consent was obtained from all respondents. The study protocol was approved by the Review Board of the Ministry of Health of Serbia and the Institute of Public Health of Serbia [14,24]. We analyzed data on respondents aged 20 years and older for whom data on body mass index (BMI) was available (89.6% of all interviewed respondents).

### Study variables

Demographic (age, gender, type of settlement, and marital status) and socio-economic (education and employment status) data, body weight and height, and data on the presence of chronic diseases were included in the present study. Age was categorized into three ranges: 20–44 years, 45–64 years, and ≥ 65 years [25]. The type of settlement was categorized as urban or rural, and marital status as married/living with partner or not married/divorced/widowed. Education was defined as high level (university degree), medium level (three of four years of secondary school), or low level (no education, incomplete primary school, or primary school), according to the International Standard Classification of Education [26]. Employment status was divided into three categories: employed, inactive (unable to work), and unemployed. Body weight and height were measured according to a defined protocol and used to calculate BMI (weight in kilograms divided by height in meters squared). Respondents were determined to be obese if their BMI was ≥ 30.0 kg/m^2^. The presence of chronic disease was determined with a questionnaire that asked the following question: “Have you had any of the following diseases or conditions in the previous 12 months?” The list of diseases/conditions included bronchial (including allergic) asthma, chronic bronchitis/chronic obstructive pulmonary disease (COPD)/emphysema, myocardial infarction or the long term-consequences thereof, coronary heart disease or angina pectoris, hypertension, stroke or the long-term consequences thereof, arthrosis/degenerative joint disease (excluding arthritis), deformity of the lower spine or neck or other chronic problem with the spine, diabetes mellitus, allergy (excluding allergic asthma), cirrhosis, urinary incontinence, kidney disease, depression, malignancy, and hyperlipidemia. Data on the following 12 chronic diseases were analyzed: bronchial asthma, chronic bronchitis/COPD/emphysema, myocardial infarction or the long-term consequences thereof, coronary heart disease or angina pectoris, hypertension, stroke or the long-term consequences thereof, arthrosis/degenerative joint disease (excluding arthritis), diabetes mellitus, kidney disease, depression, malignancy, and hyperlipidemia. Obesity was included in the analysis based on the results of other studies [4,10,25,27–29]. Multi-morbidity was defined as the presence of two and more chronic diseases in the same person [2,3].

### Statistical analysis

Statistical analyses were carried out separately for men and women and age groups. Continuous variables, described with means and standard deviations, as well as categorical variables, described with frequencies and percentages, were used for descriptive analysis. Prevalence rates of chronic diseases and multi-morbidity were estimated with appropriate 95% confidence intervals (CIs), which were weighted using probability-sampling weights calculated to reflect the inhabitants of the Republic of Serbia in 2011. Variance estimates and CIs were used to assess the impact on the precision of stratification and sampling weights using Taylor-series linearization techniques for complex samples. The χ^2^, Mann-Whitney U, and Kruskal-Wallis tests were used where appropriate.

In our analysis, we adopted the following inclusion criteria, which were similar to those used in a previous study [7]: prevalence of chronic diseases ≥ 1%; data on chronic diseases in binary form (0 = no disease and 1 = presence of the disease); and application of the principal components method for the extraction of factors by assuming nonparametric distribution of binary data (presence/absence of a disease) and the use of scree plots and parallel analysis for the selection of a number of factors, the Varimax orthogonal rotation method, and factor scores > 0.25 as the minimum acceptable value for a correlation that was significant from clinical and statistical standpoints, as well as the identification of at least two diseases per factor. The principal components method was applied to factor extraction, with Eigenvalues > 1. Kaiser-Meyer-Olkin was used as a measure of sample adequacy for each age and gender group. A previous analysis yielded a proportion of cumulative variance as a measure of the goodness-of-fit model. The prevalence of multi-morbidity patterns according to generated factors was calculated in respondents with multi-morbidity, as well as in the diseased population and in all respondents in order to determine the extent of multi-morbidity in the population.

All statistical analyses were carried out using SPSS v.20.0 software (SPSS Inc., Chicago, IL, USA) and STATA v.11.1 (StataCorp LP, College Station, TX, USA) with complex sampling design taken into account. Statistical significance was set at a two-sided P value < 0.05. All data were anonymized prior to access and analysis by the researchers.

## Results and Discussion

### Respondents

The study included 13,103 respondents, of which 48.1% were men and 51.9% were women. The average age of respondents was 49.4; 5,472 were in the 20–44 age group, 4,882 in the 45–64 age group, and 2,749 were ≥ 65 years old. The majority of respondents resided in urban areas, was married/living with partner, had a medium level of education, and was unemployed (Table 1).

**Table 1:** Characteristics of study respondents by age, Republic of Serbia, 2013.

Variables	All	Age 20–44	Age 45–64	Age ≥ 65	P*
	(n = 13103)	(n = 5472)	(n = 4882)	(n = 2749)	
**Age, mean (SD)**	49.4 (17.3)	32.3 (7.1)	55.0 (5.7)	73.7 (6.3)	<0.001
**Gender, n (%)**					
Men	6306(48.1%)	2761(50.5%)	2370(48.6%)	1175(42.7%)	<0.001
Women	6797(51.9%)	2711 (49.5%)	2511(51.4%)	1575(57.3%)	
**Type of settlement, n (%)**					
Urban	7840 (59.8)	3449 (63.0)	2908 (59.6)	1484 (54.0)	<0.001
Rural	5263 (40.2)	2023 (37.0)	1974 (40.4)	1266 (46.0)	
**Marital status, n (%)**					
Married/living with partner	8490 (64.8)	3143 (57.4)	3810 (78.0)	1538 (55.9%)	<0.001
Living without partner^a^	4612 (35.2)	2329 (42.6)	1072 (22.0)	1211 (44.1)	
**Education, n (%)**					
High	2314 (17.7)	1110 (20.3)	819 (16.8)	385 (14.0)	<0.001
Middle	7378 (56.3)	3698 (67.6)	2827 (57.9)	853 (31.0)	
Low	3409 (26.0)	663 (12.1)	1235 (25.3)	1511 (55.0)	
**Employment status, n (%)**					
Employed	4773 (36.4)	2859 (52.2)	1899 (38.9)	15 (0.5)	<0.001
Inactive	82 (0.6)	22 (0.4)	31 (0.6)	29 (1.1)	
Unemployed	8248 (62.9)	2591 (47.4)	2951(60.5)	2706 (98.4)	
**Number of chronic diseases, n (%)**					
0	6872(52.5)	4477 (81.8)	1929 (39.5)	466 (17.0)	<0.001
1	2708 (20.7)	673 (12.3)	1329 (27.2)	705 (25.6)	
2	1636 (12.5)	220 (4.0)	782 (16.0)	633 (23.0)	
3	988 (7.5)	63 (1.2)	451 (9.2)	474 (17.2)	
4	514 (3.9)	19 (0.3)	232 (4.8)	263 (9.6)	
5	208 (1.6)	4 (0.1)	92 (1.9)	112 (4.1)	
6	(0.8)	4 (0.1)	44 (0.9)	57(2.1)	
7	42 (0.3)	3 (0.1)	12 (0.2)	27 (1.0)	
≥ 8	29 (0.2)	7 (0.1)	10 (0.2)	12 (0.4)	

*Based on χ^2^, Mann-Whitney U, or Kruskal-Wallis test where appropriate.

^a^Unmarried, divorced, or widowed.

### Prevalence of self-reported chronic diseases and multi-morbidity

Hypertension, obesity, hyperlipidemia, coronary heart disease, and diabetes were the most common chronic diseases in the study population. The prevalence of chronic diseases increased with age in both sexes and was highest in the ≥ 65 years age group, except for hyperlipidemia and obesity in men, which were the highest in the 45–64 age group (Table 2). Multi-morbidity occurred in nearly one-third of respondents and existed in all age groups. Two or more chronic diseases were present in 5.9% of respondents aged 20–44, 33.2% of those between 45–64 years, and 57.4% of those ≥ 65 years of age. Women ≥ 65 years old had the highest multi-morbidity rate (65.0%) (Table 2), with hypertension, obesity, arthrosis/degenerative joint disease, and hyperlipidemia occurring at rates of 72.0%, 34.0%, 30.2%, and 25.5%, respectively (Table 2).

**Table 2:** Prevalence (95% CI) of self-reported chronic diseases across age and gender groups, Republic of Serbia, 2013.

	All	Men			Women				
		Age 20–44	Age 45–64	Age ≥ 65	Age 20–44	Age 45–64	Age ≥ 65	P(age)	P(gender)
Chronic disease	%(95%CI)	%(95%CI)	%(95%CI)	%(95%CI)	%(95%CI)	%(95%CI)	%(95%CI)		
**Asthma**	3.5(3.2–3.8)	1.8 (1.3–2.3)	3.2(2.5–3.9)	6.7 (5.3–8.2)	1.9 (1.4–2.4)	3.2(2.5–3.9)	6.5(5.3–7.7)	< 0.001	0.117
**Chronic bronchitis, emphysema**	4.5(4.1–4.8)	1.5 (1.1–2.0)	4.1 (3.3–4.9)	7.7 (6.2–9.3)	2.3 (1.7–2.8)	4.1 (3.3–4.9)	8.6 (7.2–10.0)	< 0.001	< 0.001
**Heart attack**	2.5(2.2–2.7)	0.4(0.1–0.6)	3.6 (2.9–4.4)	8.2 (6.7–9.8)	0.2(0.03–0.4)	3.6 (2.9–4.4)	5.3 (4.1–6.3)	< 0.001	< 0.001
**Coronary heart disease**	10.7(10.2–11.2)	1.4 (0.9–1.8)	9.5 (8.3–10.6)	23.7 (21.3–26.1)	1.5(1.0–1.9)	9.5 (8.3–10.6)	31.5 (29.2–33.8)	< 0.001	< 0.001
**Hypertension**	32.7(31.9–33.5)	9.2 (8.2–10.3)	37.2 (35.3–39.2)	54.9 (52.0–57.7)	6.7(5.8–7.6)	37.2(35.3–39.2)	72.0(70.0–74.2)	< 0.001	< 0.001
**Stroke**	1.9(1.7–2.2)	0.2 (0.04–0.4)	2.5(1.9–3.1)	4.7 (3.4–5.6)	0.3 (0.1–05)	2.5(1.9–3.1)	4.9 (3.9–6.0)	< 0.001	0.659
**Arthrosis-degenerative joint disease**	9.6(9.1–10.1)	1.4 (1.0–1.8)	6.5 (5.5–7.5)	13.4 (11.4–15.3)	2.0 (1.4–2.5)	6.5(5.5–7.5)	30.2 (28.0–32.5)	< 0.001	< 0.001
**Diabetes mellitus**	8.0(7.5–8.4)	1.5 (1.0–1.9)	9.6 (8.4–10.8)	16.4 (14.3–18.6)	2.0(1.4–2.5)	9.6(8.4–10.8)	18.4 (16.5–20.3)	< 0.001	0.010
**Kidney disease**	5.7(5.3–6.1)	2.2 (1.7–2.7)	5.1(4.2–6.0)	9.4(7.7–11.1)	3.1 (2.5–3.8)	5.1(4.2–6.0)	11.8 (10.2–13.4)	< 0.001	< 0.001
**Depression**	6.5(6.1–6.9)	2.4 (1.9–0.3)	5.7 (4.7–6.6)	6.3 (4.9–7.7)	3.9(3.2–4.6)	5.7(4.7–6.6)	13.3(11.6–15.0)	< 0.001	< 0.001
**Malignancy**	1.5(1.30–1.7)	0.5 (0.2–0.8)	1.4 (0.9–1.9)	2.4(1.5–3.2)	0.7(0.4–1.0)	1.4(0.9–1.9)	2.9 (2.0–3.7)	< 0.001	0.004
**Hyperlipidemia**	13.8(13.2–14.4)	4.5 (3.8–5.3)	16.8 (15.3–18.3)	15.6 (13.6–17.7)	4.2(3.4–4.9)	16.8(15.3–18.3)	25.5(23.4–27.7)	< 0.001	< 0.001
**Obesity**	22.5(21.8–23.2)	15.8(14.4–17.1)	27.5 (25.7–29.3)	22.2(19.8–24.6)	10.7(9.6–11.9)	27.5(25.7–29.3)	34.0(31.7–36.3)	< 0.001	0.003
**Multimorbidity**	26.9(26.1–27.6)	5.3 (4.5–6.2)	27.7(25.9–29.5)	47.3(44.4–50.1)	6.4(5.5–7.3)	38.5(36.6–40.4)	65.0(62.6–67.3)	< 0.001	< 0.001

Multi-morbidity was defined as the presence of two or more diseases/conditions. CI, confidence interval; P (age), statistical differences between the three age groups; P (gender), differences between sexes irrespective of age group.

### Multi-morbidity patterns

In men aged 20–44 years, factor analysis of chronic diseases confirmed the presence of two factors. The first factor was multi-morbidity pattern/non-communicable, which was determined by the association between stroke, heart attack, malignancy, chronic bronchitis/COPD/emphysema, coronary heart disease, asthma, diabetes mellitus, degenerative joint disease, depression, and kidney disease. The second factor was cardio-metabolic, which included hypertension, hyperlipidemia, and obesity (Table 3). In men aged 45–64 years, the factor analysis of chronic diseases revealed four factors: factor 1 included a respiratory pattern, clustered chronic bronchitis/COPD/emphysema, and asthma; factor 2, cardio-metabolic and clustered hypertension/obesity/diabetes/hyperlipidemia; factor 3, aggregate, combined degenerative joint disease, depression, kidney disease, stroke; and malignancy; and factor 4, cardio-vascular, combined heart attack, coronary heart disease, and hyperlipidemia (Table 3). In men ≥ 65 years, the factor analysis revealed four factors: factor 1, respiratory, clustered chronic bronchitis/COPD/emphysema/asthma; factor 2, cardio-metabolic and coronary heart disease, as well as diseases encompassed by factor 2 in men aged 45–64 years (obesity, hypertension/diabetes/hyperlipidemia); factor 3, cardiovascular, combined hyperlipidemia, heart attack, coronary heart disease, malignancy, and stroke; and factor 4, mechanical/mental/metabolic diseases, which was similar to those included as factor 3 in men aged 45–64 years, but with hypertension instead of malignancy (Table 3).

**Table 3:** Factor scores for chronic diseases in male respondents across age groups, Republic of Serbia, 2013.

	Age 20–44		Age 45–64				Age ≥ 65			
Chronic disease	Factor 1	Factor 2	Factor 1	Factor 2	Factor 3	Factor 4	Factor 1	Factor 2	Factor 3	Factor 4
Asthma	0.497		0.855				0.843			
Chronic bronchitis, emphysema	0.537		0.859				0.834			
Heart attack	0.788					0.812			0.723	
Coronary heart disease	0.529					0.768		0.262	0.549	
Hypertension		0.739		0.657				0.591		0.290
Stroke	0.831				0.430				0.410	0.305
Arthrosis-degenerative joint disease	0.425				0.620					0.544
Diabetes mellitus	0.436			0.593				0.559		
Kidney disease	0.353				0.586					0.538
Depression	0.382				0.601					0.692
Malignancy	0.672				0.319				0.411	
Hyperlipidemia		0.619		0.507		0.315		0.425	0.377	
Obesity		0.598		0.596				0.628		
KMO; P	0.829; <0.001		0.656;<0.001				0.616; <0.001			

KMO, Kaiser*-*Meyer*-*Olkin measure of sampling adequacy.

In women aged 20–44 years, the factor analysis revealed three factors: the first—non-communicable multi-morbidity pattern—was based on the association between heart attack, malignancy, stroke, coronary heart disease, depression, degenerative joint disease, and diabetes. The second factor—cardio-metabolic—included hypertension, hyperlipidemia, obesity, diabetes, and coronary heart disease. The third factor, respiratory, combined chronic bronchitis/COPD/emphysema, asthma, and kidney disease (Table 4). In women aged 45–64 years, the following three factors were identified: factor 1, cardio-metabolic pattern, combined hypertension/obesity/diabetes/hyperlipidemia, and coronary heart disease; factor 2, respiratory pattern, combined chronic bronchitis/COPD/emphysema, asthma, kidney disease, and depression; and factor 3, cardiovascular pattern, combined heart attack/stroke/coronary heart disease, and depression (Table 4). In women aged ≥ 65 years, four factors were identified: factor 1, respiratory, clustered chronic bronchitis/COPD/emphysema, and asthma; factor 2, cardio-metabolic, which was similar to factor 2 for women aged 20–44 years and factor 1 for women aged 45–64 years; factor 3, which clustered heart attack/coronary heart disease/stroke, and kidney disease; and factor 4, mechanical-mental-metabolic, in which degenerative joint disease, depression, kidney disease, and diabetes showed a negative correlation (Table 4).

**Table 4:** Factor scores for chronic diseases in female respondents across age groups, Republic of Serbia, 2013.

	Age 20–44			Age 45–64			Age ≥ 65			
Chronic disease	Factor 1	Factor 2	Factor 3	Factor 1	Factor 2	Factor 3	Factor 1	Factor 2	Factor 3	Factor 4
Asthma			0.753		0.786		0.813			
Chronic bronchitis, emphysema			0.776		0.810		0.795			
Heart attack	0.737					0.666			0.786	
Coronary heart disease	0.455	0.274		0.319		0.458			0.529	
Hypertension		0.691		0.692				0.486		
Stroke	0.586					0.554			0.463	
Arthrosis-degenerative joint disease	0.279	0.276		0.334						0.668
Diabetes mellitus	0.341	0.522		0.602				0.630		-0.326
Kidney disease			0.376		0.268				0.290	0.290
Depression	0.400				0.260	0.424				0.662
Malignancy	0.628									
Hyperlipidemia		0.616		0.490				0.564		
Obesity		0.605		0.609				0.548		
KMO; P	0.752; <0.001			0.696; <0.001			0.643; <0.001			

KMO, Kaiser*-*Meyer*-*Olkin measure of sampling adequacy.

In every age group, the respiratory pattern affected a similar proportion of men and women. The non-communicable pattern was observed in men and women only in the 20–44 age group, with higher prevalence among men. This pattern was most frequently observed in the diseased population with multi-morbidity (29.7% of men and 13.3% of women).

The prevalence and patterns of multi-morbidity in both sexes with respect to the total study population, diseased population, and diseased population with multi-morbidity are shown in Table 5. The cardio-metabolic pattern had the highest prevalence in both sexes and all age groups. In the middle-aged group (45–64 years), the rate was 3.6 times higher in males and 2.6 times higher in females with multi-morbidity as compared to the general population. The most frequently detected pattern among male respondents aged ≥ 65 years was the cardiovascular type; the rate was 2.1 times higher in males in the diseased population with multi-morbidity and 1.3 times higher in men in the diseased population than in the general population. Nearly one-fifth of women aged ≥ years 65 in the diseased population and one-third of women in the diseased population with multi-morbidity exhibited the mechanical/mental/metabolic pattern.

**Table 5:** Prevalence of multi-morbidity patterns across gender and age groups, Republic of Serbia, 2013.

**Factor**	**Respondents aged 20–44**											
	**MMB-P (%) in the study population**				**MMB-P (%) in diseased population**				**MMB-P (%) in the diseased population with multi-morbidity**			
	**Men**		**Women**		**Men**		**Women**		**Men**		**Women**	
**Factor1**	Non-communicable	1.6	Non-communicable	0.8	Non-communicable	8.9	Non-communicable	4.6	Non-communicable	29.7	Non-communicable	13.3
**Factor2**	Cardio- metabolic	3.8	Cardio-metabolic	3.9	Cardio-metabolic	20.9	Cardio-metabolic	21.1	Cardio-metabolic	70.3	Cardio-metabolic	60.7
**Factor3**			Respiratory	1.7			Respiratory	9.5			Respiratory	26.0
	**Respondents aged 45–64**											
**Factor1**	Respiratory	3.8	Cardio-metabolic	20.5	Respiratory	7.0	Cardio-metabolic	30.8	Respiratory	13.7	Cardio-metabolic	53.2
**Factor2**	Cardio-metabolic	10.8	Respiratory	6.5	Cardio-metabolic	20.1	Respiratory	9.7	Cardio-metabolic	39.2	Respiratory	16.8
**Factor3**	Aggregate	6.7	Cardiovascular	11.4	Aggregate	12.4	Cardiovascular	17.1	Aggregate	24.3	Cardiovascular	29.6
**Factor4**	Cardiovascular	6.3			Cardiovascular	11.6			Cardiovascular	22.8		
	**Respondents aged ≥65 years**											
**Factor1**	Respiratory	7.6	Respiratory	9.4	Respiratory	10.1	Respiratory	10.6	Respiratory	16.0	Respiratory	14.5
**Factor2**	Cardio-metabolic	14.0	Cardio-metabolic	21.6	Cardio-metabolic	18.5	Cardio-metabolic	24.3	Cardio-metabolic	29.5	Cardio-metabolic	33.2
**Factor3**	Cardiovascular	13.5	Cardiovascular	12.3	Cardiovasular	18.0	Cardiovascular	13.8	Cardiovascular	28.7	Cardiovascular	18.9
**Factor4**	Mechanical/mental/metabolic	12.2	Mechanical/mental/metabolic	21.0	Mechanical/mental/metabolic	16.2	Mechanical/mental/metabolic	23.7	Mechanical/mental/metabolic	25.8	Mechanical/mental/metabolic	32.3

MMB-P (%), Prevalence of multi-morbidity patterns generated by factors

We found that 26.9% of Serbians had two or more chronic diseases. Other studies have reported variable prevalence of multi-morbidity, which is likely due to sample type (general population, primary care practices, etc.), source of data, data collection method, observed age groups, diagnoses that were considered, and study population [30–33]. The prevalence of multi-morbidity determined in this study was population-based but consistent with previous reports, indicating that it accurately reflects the health status of the Serbian population. Our analysis revealed that the prevalence of multi-morbidity increased with age (65% of women and almost 50% of men 65 years or older). These findings are in agreement with the results of many other studies [8,25,28,29,31, 34–36]. We also showed that 33% of respondents aged 45–64 years had two or more diseases. It has been suggested that multi-morbidity must be examined in different age groups in order to fully understand its complexities [28]. This is supported by the fact that in our study, 6% of respondents aged 20–44 years had two or more diseases. We also observed that nearly all chronic diseases (except for asthma, stroke, and diabetes) were more prevalent in women in the ≥ 65 years age group, indicating that special attention should be focused on the care of not only the elderly, but of women in general, as they are more likely to develop multi-morbidity [28]. A systematic review of the methodology used in studies of multi-morbidity patterns has revealed differences in approaches (i.e., methodological variability) across studies, including statistical procedures (factor or cluster analysis); three types of patterns have been defined that are linked to a combination of cardiovascular and metabolic diseases, mental health problems, and musculoskeletal disorders [8].

Disease patterns in Serbian adults identified in our study included non-communicable, cardio-metabolic, respiratory, cardiovascular, aggregate, and mechanical/mental/metabolic. Non-communicable pattern was observed in both genders but only in the 20–44 years age group, and consisted of various chronic diseases (cardiovascular, malignancy, depression, and arthrosis/degenerative joint disease). In men, this pattern also included respiratory and kidney diseases. Most diseases that clustered in this pattern have common risk factors. For instance, the predominant risk factors for cardiovascular disease and some cancers are the use of tobacco, physical inactivity, low fruit and vegetable intake, and high cholesterol [37,38]. Being overweight or obese can lead to metabolic changes and increase the risk of non-communicable diseases, including heart disease and type 2 diabetes [37–38]. Smoking is the major causative factor for COPD [39]. Risk factors for chronic kidney disease include diabetes, high blood pressure, heart disease, smoking, obesity, high cholesterol, and age 65 years or older [40]. Such pattern have not been previously reported. We surmise that despite its low prevalence in the general population, its presence in one-third of men and in one of 10 women aged 20–44 years with multi-morbidity is significant. In addition to the above-mentioned risk factors, other factors that potentially contribute to the co-occurrence of these diseases must be considered.

The disease group—which comprised men and women in all age groups and included hypertension, diabetes, obesity, and hyperlipidemia—showed the typical cardio-metabolic pattern described by other studies [4,10,25,27–29]. The group of authors claimed that obesity, which exists at an early age, can activate the immune system in both men and women and trigger a so-called metabolic syndrome [7]. Our findings provide support for this hypothesis, given that in men with this pattern, diabetes and coronary heart disease did not appear until the ages of 45 and 65 years, respectively. Furthermore, coronary heart disease as well as arthrosis/degenerative joint disease within this pattern were observed only in women aged 65 years and older. The respiratory pattern was present in both genders as asthma and COPD; it also included kidney disease and depression in young and middle-aged women, respectively. However, our factor scores for kidney disease and depression were close to the cut-off value. Earlier studies suggested that COPD is associated with systemic abnormalities, such as renal and hormonal imbalance [41,42], while another report indicated that depression is common in COPD patients [43]. Persistent asthma has also been linked to an increased risk of chronic kidney disease independent of obesity, diabetes, hypertension, and other well-established risk factors [44,45]. In Serbia in 2006, the most prevalent co-morbidities for COPD were hypertension, dyslipidemia, chronic renal diseases, and anxiety/depression [17]. Additional studies are needed to determine the mechanisms that link these diseases as a pattern. The cardiovascular pattern consisted of stroke, myocardial infarction, and coronary heart disease, and occurred starting at middle age in both men and women. This can be explained by the commonalities in the pathophysiological processes that lead to atherosclerosis. However, in men in the middle and oldest age groups, hyperlipidemia as well as malignancy were also included in this pattern. This can be partly explained by the fact that hyperlipidemia is a risk factor for cardiovascular diseases and some forms of cancer [37]. On the other hand, the cardiovascular pattern in women included kidney disease and depression; the association between these diseases has been previously described [46–49]. The mechanical/mental/metabolic pattern was found only in the oldest age category and included arthrosis/degenerative joint disease, kidney disease, and depression, as demonstrated by other studies [50–53]. The sixth pattern—termed aggregate—was complex, presenting only in middle-aged men and consisting of depression, kidney disease, arthrosis/degenerative joint disease, stroke, and malignancy; it is difficult to compare this with aggregate pattern that was previously reported, which included seven physical conditions [28]. What is clear is that arthrosis/degenerative joint diseases and depression had the highest factor scores within this pattern (0.620 and 0.601, respectively), indicating that this type of aggregate clustering merits further investigation.

### Strengths and limitations

This is the first study to address multi-morbidity patterns in the Serbian adult population. Its main strengths were the large representative sample and the statistical methodology that was used. Exploratory factor analysis was the preferred method for exploring statistical significance among stable disease clusters in one study; without inferential statistics as the foundation for analytical choices made for this method, the authors based their decisions on a set of rules and recommendations typical for social sciences [7], as described by others [54]. It is worth noting that our study met most of these standards and is self-explanatory with regards to the applicability of our findings. As in previous studies [4,10,25,27–29], we reduced the minimum factor score to 0.25 so as to increase the probability of observing a greater number of apparently random associations. Finally, the goodness-of-fit values for the models, as expressed as a percentage of the accumulated variance and sampling adequacy, were above the acceptable lower limits [4,10,25,27–29,54].

This study also had several limitations. Firstly, the use of cross-sectional survey data precluded the determination of causal relationships between analyzed variables. Secondly, the accuracy of chronic disease self-reporting depends on various factors, such as knowledge of the health problem, consequences on everyday life, willingness to report the problem, and frequency of visits to health care services [55]. Thirdly, all diseases included in our study had equal weight in the principal component analysis, since the questionnaire in the health survey did not include questions on the severity or duration of a chronic disease. An association between recent acute illness and disease reporting was assumed, since people may be more likely to seek medical help and learn more about their illnesses, which would affect the reporting of chronic diseases. Lastly, only a limited number of chronic diseases (i.e., 13) was examined due to availability of data. Nonetheless, our findings expand the current knowledge of multi-morbidity in the Serbian population.

## Conclusions

Multi-morbidity is a common occurrence in adults in Serbia, especially among the elderly. While several of the observed multi-morbidity patterns may be explained by underlying pathophysiological processes, some require further investigation. Recognizing the complexity of multi-morbidity in Serbia is of great importance from both clinical and preventive perspectives given that it affects one-third of the population and may require adjustment of the healthcare system to address the needs of affected individuals.
